# Mechanisms and implications of sex differences in cardiac aging

**DOI:** 10.20517/jca.2022.01

**Published:** 2022-03-16

**Authors:** Aykhan Yusifov, Kathleen C. Woulfe, Danielle R. Bruns

**Affiliations:** 1 Kinesiology & Health, University of Wyoming, Laramie, WY 82071, USA; 2 Division of Cardiology, University of Colorado Anschutz Medical Campus, Aurora, CO 80045, USA; 3 Division of Geriatric Medicine, University of Colorado Anschutz Medical Campus, Aurora, CO 80045, USA; 4 Wyoming WWAMI Medical Education, University of Wyoming, Laramie, WY 82071, USA

**Keywords:** Cardiac, aging, sex differences, fibrosis, adrenergic desensitization

## Abstract

Aging promotes structural and functional remodeling of the heart, even in the absence of external factors. There is growing clinical and experimental evidence supporting the existence of sex-specific patterns of cardiac aging, and in some cases, these sex differences emerge early in life. Despite efforts to identify sex-specific differences in cardiac aging, understanding how these differences are established and regulated remains limited. In addition to contributing to sex differences in age-related heart disease, sex differences also appear to underlie differential responses to cardiac stress such as adrenergic activation. Identifying the underlying mechanisms of sex-specific differences may facilitate the characterization of underlying heart disease phenotypes, with the ultimate goal of utilizing sex-specific therapeutic approaches for cardiac disease. The purpose of this review is to discuss the mechanisms and implications of sex-specific cardiac aging, how these changes render the heart more susceptible to disease, and how we can target age- and sex-specific differences to advance therapies for both male and female patients.

## INTRODUCTION

Cardiovascular diseases (CVD) are the leading cause of death worldwide^[1]^, with millions of individuals affected by CVD each year, making it a burdensome and costly public health problem. While aging impairs the cardiovascular system and is the dominant risk factor for CVD, CVD is not only a disease of the elderly. Rather, CVD is emerging in younger adults more often^[2]^. However, most of the research efforts on prevention and treatment of CVD ignore age and have focused on the development of interventions that target “traditional” CVD risk factors such as obesity, hypertension, and diabetes. While these risk factors are undoubtedly important and research efforts to understand them are critical to advance knowledge, more work is needed to understand the impact of age on the development of CVD, given the robust correlation between the development of disease and advanced age.

Aging is a complex biological process characterized by epigenetic alterations, genomic instability, cellular senescence, and mitochondrial dysfunction, amongst other cellular changes. Although there is substantial inter-individual variability in the aging process, it is clear that sex differences are present in aging. For example, women live longer than men, consistent with lower biological ages as assessed by molecular biomarkers^[3]^. Nonetheless, women are frailer and have worse health at the end of life^[3–5]^. Substantial clinical data demonstrates widespread sex differences with respect to cardiovascular structure and function. However, at present, there is relatively limited information on molecular mechanisms of sex-specific differences in cardiac aging and how sex-specific differences in the heart interact with the aging process. The reason for this lack of knowledge may be rooted in the long tradition of male-biased research^[6]^ and some of the complications in including women and female animals due to hormonal fluctuations. Here we attempt to summarize what is known of cardiac aging and sex differences that contribute to distinct cardiac aging in men and women.

## CARDIAC AGING IN MEN AND WOMEN

### The aging heart

Aging leads to deterioration of cardiac structure and function in both men and women^[7]^. Although the mechanisms are not fully clear and are multifactorial, an important contribution to increased risk of CVD with advanced age lies in greater time for exposure to injurious stimuli, such as hypertension, metabolic stress, or ischemia, over the life course. Additionally, the heart loses its capacity for repair, meaning that with repeated injury, the cumulative burden of stress is elevated, increasing the risk of disease^[8]^. Thus, it makes sense that older patients would have greater impairment of cardiac reserves and elevated risk of disease.

With advanced age, the heart becomes hypertrophic, defined as abnormal enlargement, or thickening, of the heart muscle^[9]^, largely because of an increase in ventricular myocyte size^[10]^. Elevated collagen levels and non-enzymatic cross-linking render collagen stiffer and also contribute to the ventricular thickness and tension^[11,12]^. This fibrotic process impairs ventricular function and reserve^[13,14]^, resulting in impaired diastolic function^[15]^. Systolic function is typically preserved in healthy aging; however, systolic reserve is often diminished, resulting in a heart unable to increase contraction to meet metabolic demand. The mechanisms which underlie these changes in age-related cardiac function will be discussed in more detail below, with attention to the sex differences that underlie these changes in structure and function.

### Sex differences in the aging heart and age-related cardiac disease

**Figure 1.**
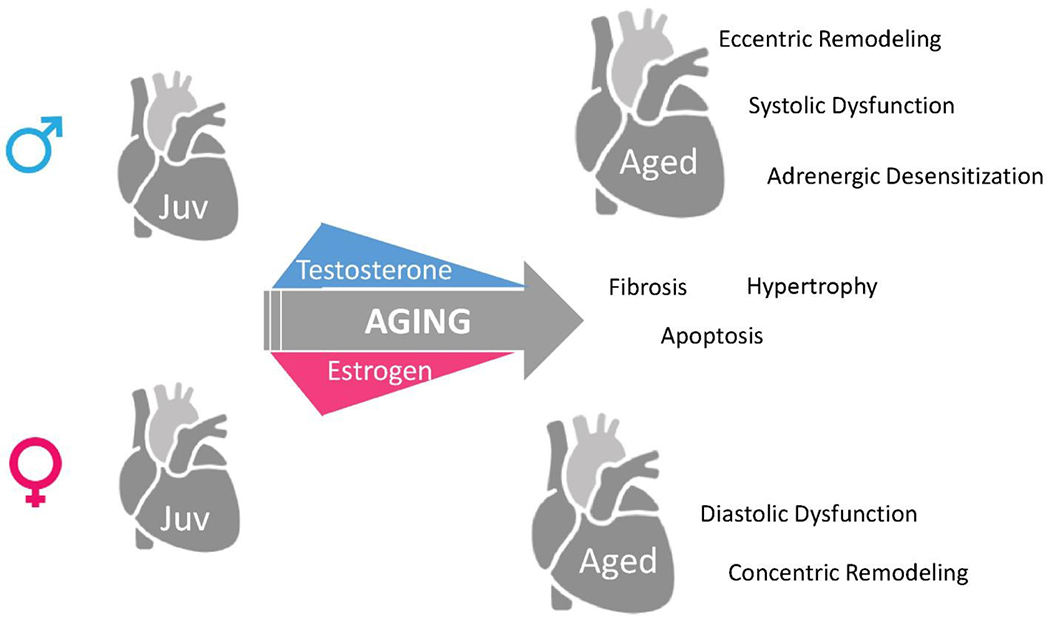
Sex-specific cardiac aging in male and female with respect to changes in major sex hormones testosterone and estrogen. While aging is characterized by ventricular hypertrophy, fibrosis, and changes in ventricular function, several mechanisms are more pronounced in the male heart compared to female. For example, the aged male heart demonstrates eccentric remodeling, systolic dysfunction, and lower adrenergic sensitivity as opposed to aged female heart, which demonstrates diastolic dysfunction and concentric remodeling. While some of these changes likely coincide with temporal changes in sex hormones, others are likely regulated by non-hormonal changes, or occur via different temporal patterns in the male and female heart.

Sex-related differences in cardiac structure and function have been reported in several clinical studies^[16–18]^. Healthy women and men have different chamber dimensions and functions, even after indexing for body size. On average, females have a smaller left ventricular (LV) chamber with approximately 10% less LV mass than males, even after indexing for body size by body surface area^[19]^. Since women have smaller LV chambers and accordingly lower stroke volumes, a higher resting heart rate maintains a similar cardiac output to men. Women also have higher systolic and diastolic LV stiffness than men at a given age, and these differences are more prominent with aging, where steeper increases in LV stiffness are seen in women compared to men^[20]^. Cardiac contractility is well preserved in women but declined in men after age 50^[21]^. These sex differences in function with age are also demonstrated pre-clinically, where animal studies show that systolic function declines with age in males but not in females^[22,23]^. Sex differences in the aged heart are also evident in cellular studies. Male cells contract more strongly and rapidly than female cells^[24,25]^, driven likely both by differences in sarcomeric protein and calcium-handling function^[24]^. Even though the number of cardiomyocytes is similar between sexes at birth^[26]^, female cardiac myocytes are less likely to undergo apoptotic cell death compared to male myocytes, resulting in an elevated risk for cardiac myocyte loss in male hearts^[27]^. Indeed, this observation is supported by a recent single-cell sequencing study which reported that human female hearts contain a significantly higher percentage of ventricular cardiomyocytes than male hearts at middle to early older age^[28]^. Together, current evidence clearly shows sex-specific differences in cardiac aging at the cellular, anatomical, and functional levels [Figure 1].

Sex differences in cardiac aging likely contribute to different age-related cardiac pathogenesis observed in clinical populations [Table 1]. Older men are more likely than older women to develop heart failure with reduced ejection fraction^[29]^. Women, on the other hand, are more likely to develop heart failure with preserved ejection fraction (HFpEF)^[30,31]^, characterized by increased wall thickness and diastolic dysfunction with little or no reduction in ejection fraction. Men are more prone to ventricular arrhythmias^[32,33]^. Men are also at higher risk for coronary heart disease at younger ages^[34]^, but women surpass men with age, and experience worse outcomes with myocardial ischemia, resulting in higher mortality and poorer quality of life^[35–37]^. In valvular heart disease, degenerative mitral regurgitation impacts a significant proportion of elderly women, particularly those with comorbidities^[38]^. While sex differences in cardiac physiology likely contribute to these clinical differences (discussed below), risk factors also vary between aged men and women, which contribute to different disease pathogenesis. For example, obesity, hypertension, and diabetes are highly prevalent in women with HFpEF, while the underlying cause of heart failure in men tends to be ischemia and coronary artery disease^[39]^. The contributions of these risk factors to the sex-specific development of cardiac aging are still being elucidated.

### Sex differences in cardiac fibrosis in the aging heart

**Figure 2.**
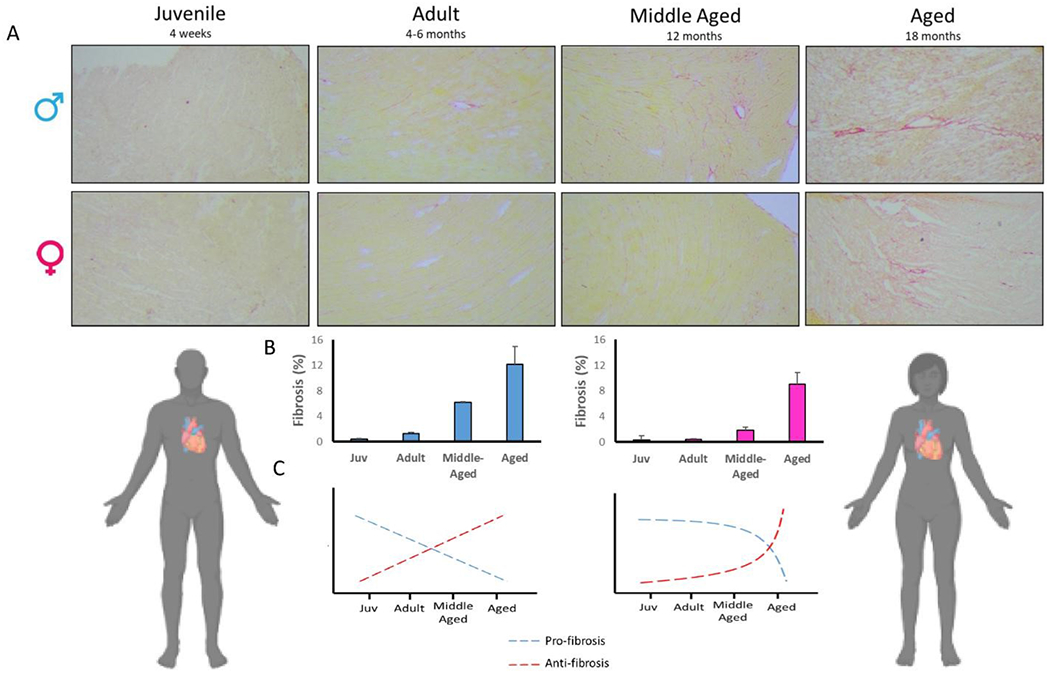
Fibrosis in the male and female heart across the life course. Collagen accumulation was assessed by picro-sirius red in LV in mice from 4 distinct age groups: juvenile (Juv; 4 weeks), adult (4-6 months), middle-aged (12 months), and aged (18 months) mice of both sexes. Quantification of fibrosis demonstrates that fibrotic content increases earlier in life for males, while females show relatively delayed fibrosis later in life. (A) Representative images; (B) quantification of fibrosis content in male and female samples. In male LV, fibrotic content was significantly higher in adult, while in female, fibrosis was not significantly elevated until middle age. *n* = 3/group; (C) expression of pro- and anti-fibrosis genes occurs in a sex-dependent manner with aging. Blue: male; pink/red: female.

Both aging and diastolic dysfunction are characterized by elevated ventricular stiffness and deposition of extracellular matrix (ECM) proteins, thus cardiac fibrosis has been pursued by several research groups^[40]^ as a mechanism of age-related heart disease. Indeed, fibrosis is associated with ventricular compliance and impaired passive filling of the LV^[41,42]^. Age-related alterations in cardiac ECM are sex-dependent, with aged male rat hearts more fibrotic than female hearts^[43]^. In human imaging-based studies, myocardial fibrosis is more pronounced in the aging male heart than the female heart^[44,45]^. While the expression of collagen in human hearts did not differ between sexes, regulators of collagen metabolism differed between sexes^[46]^. Collagen types I and III were lower in young women than young men, but with age, the trend reversed, and women expressed higher collagen I and III compared to men^[47]^. Recently, it was reported that collagen I is the predominant type in the aged male heart, whereas collagen III was the main component in the aged female mouse heart^[48]^. Moreover, the authors correlated these sex-specific differences to sex-specific regional deposition of fibrosis, with males being more likely to undergo apoptosis and concomitant reactive interstitial fibrosis compared to females. In line with these studies, unpublished data from our lab suggests that the development of fibrosis with aging occurs in a temporally distinct manner in male versus female mice. We assessed collagen accumulation by Picro-Sirius red staining in the LV in mice from 4 distinct age groups: juvenile (4 weeks), adult (4-6 months), middle-aged (12 months), and aged (18 months) [Figure 2].

Quantification of fibrosis showed that fibrotic content increases earlier in life for males, while this process was temporally delayed in females. When we analyzed the expression of key pro- and anti-fibrosis genes, we found that pro-fibrosis genes are upregulated in younger hearts of both sexes, and gradually decline with aging. Specifically, the expression of collagen I was higher in juvenile and adulthood and decreased in the aged female heart. In contrast, male hearts showed a decline in collagen I expression after juvenile-hood in mice^[49]^. On the other hand, the expression of anti-fibrotic genes gradually decreased with age, supporting the notion that accumulation of fibrous connective tissue in cardiac ECM is caused by slower removal of ECM components rather than due to increased deposition of fibrotic proteins into the matrix. These data suggest that reduced collagen degradation may be more important than increased *de novo* collagen synthesis in the pathogenesis of aging-associated fibrosis in a sex-specific manner. While activation of pro-fibrotic genes is considered to be a primary pathway for the development of fibrosis, newly emerging data consider disruption of anti-fibrotic pathways as also essential in this process. It appears that the fibrotic process occurs via different temporal trajectories by sex. Future studies which utilize life-course approaches, and/or aim to understand the differences in animals of juvenile and middle ages will yield insight into the temporal nature of these sex-specific differences in cardiac aging. Understanding the mechanisms by which male and female hearts become fibrotic with advanced age is important for the identification of anti-fibrotic therapies- a large unmet clinical need.

### Sex differences in aging at the cardiac myofilament

Even though whole cell contraction and relaxation are reliant on coordination from a myriad of regulatory processes in cardiomyocytes, the most basic contractile unit of the cardiomyocyte is the sarcomere. Sarcomeric protein interactions underlie contraction and relaxation, and thereby different protein isoforms or post-translational modifications on specific residues of sarcomeric proteins lead to significant differences in duration, rates, and intensity of contraction and relaxation. Simplistically, identifying key differences that occur at the level of the sarcomere between males and females as they age will provide the basis to understand larger, whole organ nuances and thereby provide therapeutic targets to modify aging-induced alterations.

It is clear that myofilament function is altered with age both in humans and animal models^[50–58]^ but much like other cellular systems in aging, there is high heterogeneity in aging myofilament proteins. While conclusive functional changes are complicated by differences in animal models, age of the models, as well as differences in techniques, aging seems to modify passive stiffness, force generation, and calcium sensitivity, as well as prolong relaxation of the myofilament. However, it is clear that nuances in experimental design as well as, importantly, sex and health of the models warrant careful investigation and comparison. With this in mind, a study by Kane *et al.*^[54,59]^ demonstrates the importance of incorporating a frailty assay or some assessment of whole-body aging as heterogeneity in biological aging clearly exists and likely confounds conclusions about myofilament modifications due to age. Moreover, this particular study makes it clear that the markers providing important information about aging may differ by sex since male animals had a significant correlation in their frailty scores to key modifications in their myofilament proteins, whereas female animals did not.

Of note, aging can induce altered myofilament function through either expression of different isoforms or through differential post-translational modifications. Nance *et al.*^[60]^ reported that sarcomere lengthening is impaired in aged cardiomyocytes, which alters the length-dependent activation. One sarcomeric protein that regulates sarcomere length and contributes, in part, to the dynamics of sarcomeric lengthening is titin. Interestingly, a recent study determined that in male mice or humans, titin isoforms were not altered with age. However, phosphorylation at specific sites on titin were differentially modified in male mice^[61]^. Similarly, phosphorylation of serine 44 of cardiac troponin I is elevated, and contractile function decreased in aging rats^[57]^. Moreover, numerous reports suggest that myofilament proteins are differentially modified in males and females with age^[54]^. It is clear that sex hormones differentially impact myofilament function^[62–69]^. However, reports are conflicting with the overall effect of estradiol itself on myofilament function and modifications. Most notably, several studies demonstrate increased calcium sensitivity in response to acute loss of estradiol or low estradiol levels. However, chronic loss of estradiol leads to decreased myofilament calcium sensitivity. These contrasting reports suggest that changing estrogen levels at pivotal aging events such as perimenopause represent a different regulatory milieu than pre-menopause or even menopause. Decreased testosterone also has been shown to modify myofilament function, inducing prolonged relaxation and diastolic dysfunction in aged animals^[62]^. In line with this, orchiectomized male rats demonstrate decreased active force and slower cross-bridge cycling with higher expression of beta-myosin heavy chain and lower phosphorylation of key sarcomeric proteins^[69]^. However, an important consideration is that the majority of the sex hormone studies have been completed in adult or young animals and not in old animals. Therefore, while it is clear that estrogen and testosterone impact myofilament post-translational modifications, determining how age itself impacts myofilament along with hormonal changes is critical.

## MECHANISMS OF SEX DIFFERENCES IN CARDIAC AGING

Sex differences in cardiac disease have long been attributed to estrogen, a hypothesis which largely stems from the loss of cardioprotection in women following menopause. While estrogen undoubtedly plays a significant role in the cardiac disease phenotype between men and women, the contribution of other sex hormones as well as non-hormonal mechanisms have emerged as critical regulators of cardiac disease and will be the focus of this section.

First, to help guide our discussion, we begin with a conceptual framework for understanding the basis of sex differences. This model was put forth by Arnold^[70]^ to guide the study of sex-based differences in physiology and disease. This framework suggests that there are three causes of sex differences. First are the effects that are due to activation of gonadal steroid hormones, such that shortly after removing them, [i.e., by Ovariectomy (OVX) and orchiectomy] the effects are attenuated. Several effects of gonadal steroid hormones persist following gonadectomy, and these are referred to as long-term organizational effects of hormones- i.e., the persistence of internal and external genitalia. Lastly, even following gonadectomy, there remain differences that cannot be explained due to steroidal hormones, but are rather due to the effects of sex chromosome genes acting outside the gonads. This framework leads to a relatively standard strategy for understanding sex-based differences and identifying sex-based factors-1) remove the gonads and quantify phenotypic changes. If they are present, identify which hormone (estrogens, testosterone, progesterone) drives the phenotype. If sex differences persist following gonadectomy, then organizational effects and non-hormonal effects should be studied. While this paradigm is simple and straightforward and certainly provides mechanistic insight, to date, the bulk of what is known about sex differences has focused predominantly on estrogen, with sex chromosome effects rarely investigated. We propose that a comprehensive analysis of sex hormone and chromosome-regulated mechanisms will yield a greater understanding of sex differences in cardiac aging and disease.

### Sex hormone-mediated differences in cardiac aging

In premenopausal women, 17β-estradiol (E2) produced by the ovaries is the primary circulating estrogen. High concentrations of E2 act primarily as an endocrine factor on distal tissues. Serum estradiol concentrations are low in adolescence and increase at menarche. In adult women, estradiol fluctuates with the menstrual cycle, ranging from 100 pg/mL in the follicular phase to about 600 pg/mL at ovulation. Estradiol is high during pregnancy, and then after menopause, concentrations fall to similar values or lower to those in age-matched men (5 to 20 pg/mL). Following menopause and in men, extragonadal sources are responsible for the low levels of E2 production, largely acting in paracrine roles. While aging female rodents do not undergo true menopause, they do become reproductively incompetent or senescent with advanced age, a state referred to as estropause. Estropause is characterized by persistently lower estrogen, varying length of the estrous cycle, with eventual cessation of cyclicity around 12-14 months of age^[71]^. Ovariectomy of aged animals supports the loss of estrogen with aging, as animals do not undergo changes in metabolic function with ovarian estrogen removal, nor do significant changes in cardiac phenotype occur with removal of the ovaries^[72]^. However, OVX and aging are not synonymous. While they both are characterized by loss of ovarian estrogen, as discussed above, the aged heart is phenotypically distinct from the adult-both in the basal as well as the stressed state. Thus, we propose that future work dissect the impact of estrogen from aging on cardiac function using approaches which uncouple biological age from ovarian estrogen status.

Most data regarding estrogen signaling refers to E2. E2 is inversely associated with cardiovascular disease events in postmenopausal women, with women maintaining high estrogen levels having lower heart disease risk^[73]^. A wealth of data has demonstrated beneficial effects of E2 treatment on the heart, including reduced fibrosis, attenuated oxidative stress, improved mitochondrial function, and attenuation of cardiac hypertrophy^[74]^. Estrogenic effects in the heart are due to signaling through estrogen receptors α and β (ERα and ERβ) as well as G-protein-coupled ER (GPER). It is well-accepted that the myocardium is responsive to circulating androgens and estrogens, due to the expression of ERα and ERβ in the myocardium, likely in multiple cell types including myocytes and cardiac fibroblasts^[75]^. E2 binds ER, the complex internalizes, translocates to the nucleus, and activates transcription of estrogen-responsive genes. In addition to the protection afforded by E2 *in vitro* and in pre-clinical models of cardiac disease, the beneficial role of E2 is also supported by studies that show depletion of ovarian estrogen by OVX reverses the protective effects of E2^[76]^. These gain and loss of E2 studies, along with the epidemiology data showing clear loss of cardiovascular disease protection with menopause, led to the early hypothesis that restoration of E2 with aging would reduce heart disease morbidity and mortality. However, early studies to give back E2 to the aged heart were not successful^[77]^. Aging has been suggested to diminish the ability of estrogen to be protective, and a “timing” hypothesis for estrogen therapy has emerged^[78]^. In aged spontaneously hypertensive rats, E2 delivery did not attenuate hypertrophy or molecular signatures of the failing heart, such as myosin heavy chain expression^[78]^. The authors suggested that this was in part due to not only reduced E2 synthesis but also impaired estrogen metabolism, given differences in expression of 17β-HSD, which catalyzes the reduction of weak estrogens into potent estrogens like E2. Indeed, estrogen metabolites (2-hydroxyestradiol and 2-methoxyestradiol) are also emerging as regulators of cardiac function, likely through some similar mechanisms as E2. Age-related changes in estrogen receptor and GPER expression have also been studied as a mechanism to explain the loss of cardioprotection with age. While cardiac GPER expression appears to increase with age in mice of both sexes, cardiac ERα decreases with age in females but remains unchanged in males^[79]^. Together, while it is clear that E2 is cardioprotective and loss of E2 occurs with advanced age, the mechanisms by which E2 protects the heart, how this protection declines with age, and effective therapeutic strategies for interventions against age-related declines have yet to be fully elucidated.

Testosterone decreases with advanced age, not only in men^[80]^, but also in women^[81]^. Age-dependent reductions in testosterone are also evident in aged male rodents^[82]^. Considerable evidence has emerged that low testosterone may therefore also contribute to increased risk for heart disease with aging^[73,83,84]^. The link between testosterone and heart disease is particularly strong for diseases of impaired contractility such as heart failure. The biological effects of testosterone occur through androgen receptors expressed in the heart, at least in cardiac myocytes^[85]^. Binding of androgens causes transcriptional regulation of androgen-responsive genes. However, similar to estrogen, non-genomic actions of testosterone have also been described.

Mechanistic understanding of androgens on cardiac function comes from studies of gonadectomy. In young mice, bilateral orchiectomy attenuates contractile function^[86]^. Long-term withdrawal of testosterone slows relaxation^[62]^. On the other hand, epidemiological studies of anabolic steroid users indicate that high levels of exogenous testosterone negatively impact the heart^[87]^. The long-term effects of modest or physiological levels of testosterone on the heart remain unsettled, as evidenced by the recent initiation of a clinical trial to determine the effects of long-term testosterone treatment on cardiovascular outcomes^[88]^. The impact of gonadectomy in the aging heart is not clear but likely differs from those in the young, given that gonadectomy in aged mice did not change testosterone or measures of muscle mass - findings contrary to those reported in younger male mice^[89]^.

In addition to direct androgen effects, testosterone also impacts cardiac function due to the fact that estrogen biosynthesis is dependent on testosterone availability. Testosterone can be converted to E2 by aromatase, expressed in non-gonadal tissue. While the expression of aromatase in the heart is contentious^[79,90]^, even low expression may meaningfully contribute to E2 synthesis in a setting of low systemic concentrations. E2 produced by aromatase has been speculated to act locally in a paracrine or autocrine manner, rather than as a hormone as when gonadal E2 synthesis is intact. While the cellular location of aromatase expression has not been fully described, there is some evidence that it is predominantly expressed in the coronary vasculature, with lower expression in cardiac myocytes^[91]^. To date, the expression of aromatase in cardiac fibroblasts is not known. Cardiac localized aromatase likely controls the balance between testosterone and estrogen, permitting sex steroid regulation of cardiac function. In support of this hypothesis, deletion of aromatase profoundly alters the cardiac stress response^[90]^. Understanding the contribution of aromatase to E2 synthesis in the postmenopausal and aging heart is an area ripe for future research.

As alluded to above, dissection of aging from loss of gonadal hormones with advanced age is not yet clear. That is- are age-related changes in cardiac function due to natural aging, due to age-related declines in estrogen, or both? Clinical studies of women in different phases of the menstrual cycle as well as peri-, menopausal, and postmenopausal women, suggest that while sex hormones (especially E2 and FSH) regulate arterial stiffness, the effects are largely driven by age^[92]^. In support of this hypothesis, OVX did not cause independently cause cardiac remodeling and dysfunction in rats, but rather aging resulted in diastolic dysfunction and mild systolic impairment^[93]^. Together, these findings suggest that associations between hormones and cardiovascular function are likely different at different ages or reproductive stages. These types of studies should be investigated by removal of gonads in aged mice and quantifying cardiac function in aged animals.

### Sex chromosome mediated mechanisms of cardiac aging

We recently reported significant differences in the cardiac transcriptome that are apparent before the onset of major sex hormones and sexual maturity, as well as in the hearts of reproductively incompetent aged females^[49]^. The cardiac proteome also appears to develop differently between males and females, independent of sex hormones^[94]^, given the robust differences in proteomics in mice at embryonic day E9.5, with gonadal development occurring at day Ell. When sex hormones cannot explain sex differences, either due to the persistence of sex differences post removal of gonads, it is logical to conclude that non-hormone mediated mechanisms must be contributing.

Clinical genomics studies suggest that age-related cardiac disease develops in conjunction with sex chromosomes. Polysomy of the Y chromosome is associated with elevated CVD mortality due to atherogenic lipid profiles^[95]^. In addition, women with monosomy X (Turner Syndrome) also have an elevated risk for coronary artery disease^[96]^. However, in humans, sex chromosome aneuploidy conditions (XO and XXY) are also associated with aberrant hormonal levels, making it difficult to separate chromosomal effects. To circumvent this problem, several mouse models have been generated which permit the dissection of sex chromosome differences from gonadal hormones. For the purposes of this review, we will discuss two of such models, though we refer the reader to reviews on sex hormones and chromosomes in CVD for more details^[97]^. To dissect the contributions of sex hormones from chromosomes, mice have been generated in which sex chromosomes are separate from gonadal hormones. In the case of the four core genotype (FCG) mouse, two separate mutations delete the testis-determining gene (Sry) from the Y chromosome or insert it into an autosome. This results in an XX female mouse, an XY- mouse which develops ovaries and subsequently ovarian hormones, as well as XY-Sry and XXSry transgenic mice that are gonadal males. If XX and XY mice differ despite similar hormone levels, then sex chromosomes are likely responsible for the phenotype. The XY* mouse is the other common model, where XY* mice possess the Y* chromosome, which has an aberrant pseudoautosomal region with permits crossing over with X chromosome during meiosis. This produces abnormal recombination of X and Y. Mating XY* males to XX females produces progeny that are gonadal males or females, each with one *vs*. two X chromosomes.

Given the relatively recent emergence of these models, cardiac and certainly aging studies utilizing them are sparse. In the FCG mouse model, XX mice, compared to XY mice, had more beneficial lipid profile in the form of elevated high-density lipoprotein^[98]^. The magnitude of hypertension induced by angiotensin II is greater in gonadectomized XX mice compared to XY^[99]^. In the XY* mouse model, at baseline at two months of age, all mice had a similar cardiac function, but X mice have higher vulnerability to I/R injury compared with XY* mice, due to the number of X chromosomes rather than the absence of the Y chromosome^[100]^.

Collectively, it is clear that neither hormones nor chromosomes alone are responsible for sex differences in cardiac disease and aging. Rather, the two mechanisms likely work separately as well as through overlapping or intersecting mechanisms to regulate cell and organ-level physiological and molecular differences. A recent publication using the FCG model quantified the cardiac proteins under the regulation of hormones (proteins that segregated with ovaries and testes) versus those that were chromosomally controlled (segregated with chromosomes). While the authors identified 519 under hormonal control and 159 proteins under chromosomal control, they also identified a subset that involved a combination of chromosome and hormonal control^[94]^. They speculated that these genes are regulated by both mechanisms or, could be driven by chromosomes but opposed by hormones. In the latter interpretation, male and female disease phenotypes would be similar in presentation, but the mechanism of development would vary by sex. In this case, treatment of the disease could also vary between males and females. With respect to aging, as briefly discussed above, dissecting chromosomes and age-related changes in hormones is necessary to fully elucidate sex differences in the heart. In a series of experiments aimed at understanding how gonadal hormones and chromosomes influence hypertension, adverse sex chromosome effects which contributed to hypertension were exacerbated by the removal of gonadal estrogen by OVX. That is, the interaction between the X chromosome and estrogen could contribute to hypertension in postmenopausal women^[99]^. To date, these types of investigations are incredibly limited and warranted to dissect the mechanisms of cardiac aging in male and female hearts.

## SEX DIFFERENCES IN THE CARDIAC STRESS RESPONSE VIA THE ADRENERGIC CASCADE

While sex differences in healthy aging are important, as discussed above, so are sex differences in the aged heart response to stress and/or ability to tolerate stress. The aging heart is well-characterized by a diminished stress response, resulting in elevated morbidity and mortality compared to younger animals. In young models, the female advantage is also well-characterized, with young females being at less risk of developing heart disease compared to males of similar age^[101]^. The female advantage declines with advanced age, as evidenced by a steeper rate of heart disease risk increase in women older than 50 compared to men^[102]^. However, given the assumption that sex differences disappear with estro/menopause, differences in the aged male and female heart to cardiac stress have been sparsely studied. These studies are also complicated by the diminished ability of aged cohorts to survive cardiac insult, further contributing to a lack of sex difference studies in the aging heart.

In response to cardiac stress, compensatory mechanisms are engaged in an effort to maintain cardiac function^[103]^. One of these major compensatory mechanisms is the activation of neuro-hormonal system, largely mediated by the stimulation of adrenergic receptors (AR) by catecholamines^[104,105]^. Activation of AR and downstream signaling increases calcium sensitivity and phosphorylates myofilament contractile proteins, shifting calcium affinity and contractile dynamics, in an effort to increase cardiac work. The human heart expresses two broad classes of adrenergic receptors, the α-adrenergic and the β-adrenergic families. Each of these families can be further subdivided into subclasses, but since the β-adrenergic receptor (β-AR) cascade has been the focus of active research in the context of cardiac function, we will focus on sex differences in the β-AR and changes in this cascade with advanced age.

β-AR are members of the G protein-coupled receptor (GPCR) superfamily of receptors. There are three major subtypes of β-ARs identified in the human heart, α1-, β2-, and β3-AR^[106–108]^, and of these, β1-AR and β 2-AR have earned the greatest interest due to their role in myocardial contraction. In brief, activation of the cascade starts with the binding of β-AR agonist, which causes a conformational change in the receptor. A primary effect of the β-AR is stimulation of adenylyl cyclases, multiple subtypes of which are expressed in human cardiac tissues. Adenylyl cyclases catalyze the conversion of ATP to the second messenger cAMP, which in turn binds to the regulatory subunits of protein kinase A (PKA). PKA phosphorylates serine and threonine residues on a number of proteins, thereby affecting a spectrum of cellular processes ranging from contractility to global gene expression patterns. Important PKA targets that acutely modulate myocardial contractility are β-ARs, L-type Ca^2+^ channels, the sarcoplasmic reticular Ca^2+^ ATPase inhibitory protein, phospholamban, and troponin I (TnI)^[109]^. Once the incoming signal is transduced, termination of the signal is accomplished to balance activation and deactivation. β-ARs deactivation is mainly accomplished through the actions of GPCR kinases (GRKs)^[110]^. GRK recruits β-arrestins, which uncouple the receptor from G-proteins and promote internalization and down-regulation of the receptor^[110]^.

### Adrenergic regulation of cardiac function with aging

Age-related declines in the responsiveness of adrenergic activation are well-established^[111]^. Decline in sensitivity to catecholamine stimulation has been attributed to high levels of circulating catecholamines, which lead to downregulation of β-adrenergic receptor and pathway activation^[105,112,113]^. Diminished β-adrenergic receptor sensitivity is called “β-adrenergic desensitization” and is characterized by altered ventricular inotropic reserve and exercise intolerance^[111,114]^. The underlying mechanisms have not been fully elucidated, but evidence suggests that fewer β-adrenergic receptors, other components of the β-adrenergic signaling pathway, or a combination play a role^[111,112,114,115]^. In addition to adrenergic desensitization, aging also influences the cardiac response to adrenergic-mediated therapeutics. Stimulation of AR with receptor agonists has been reported to have a deleterious effect on cardiac function in patients over the age of 65^[116]^. Although to date, no large clinical trial has specifically set out to examine β-blockade in older patients, a small uncontrolled observational study with patients with a mean age of 78 noted twice the rate of withdrawal and no improvements in symptoms of chronic heart failure^[117]^ compared to younger patients. Given the strong association of advanced age with heart disease risk, understanding the impact of age on adrenergic therapeutics such as β-blockade is warranted.

### Sex differences in the cardiac adrenergic cascade

In addition to differences with age, sex differences also exist in cardiac function in response to AR stimulation. Female patients maintain cardiac output through changes in heart rate^[118]^, while male hearts tend to utilize changes in Frank-Starling mechanisms to increase cardiac output. Animal studies also reveal sex differences in AR stimulation and cardiac performance. Data from our group in a model of isoproterenol-mediated adrenergic activation support this observation, with a significant increase in cardiac output in adult females, driven by higher heart rate alongside unchanged stroke volume. Isoproterenol also resulted in sex-specific changes in cardiac structure, function, and gene expression across age and sex^[119]^, sex differences which were differentially changed with age. Previous reports of catecholamine stimulation in humans demonstrated that adrenaline infusion in young (average age 30) and older (average age 60) adults caused similar increases in heart rate, but larger increases in stroke volume and ejection fraction in younger compared to older subjects^[120]^. Additionally, young males demonstrated a greater increase in heart rate than young females in response to adrenergic receptor stimulation. Together, these data suggest that with age, males demonstrate exacerbated decline in adrenergic-mediated function.

Despite the well-established observation that declines in adrenergic activity occur with aging and disease and that β-ARs are key pharmaceutical targets, only a few studies have investigated mechanistic sex differences, and the results of these studies have been inconsistent^[121–123]^. Gonadal hormones influence the response to the β-blockers in animal models. In a rat study, β-blocker treatment was effective only in males, but not in gondally intact females^[124,125]^. When it comes to the comprehensive assessment of the adrenergic cascade, the majority of the studies investigating sex differences have assessed the contractile response^[121,126]^ with a smaller number of studies aimed at understanding calcium handling^[127,128]^. For instance, when the expression and abundance of calcium handling proteins are compared between sexes, female rats showed significantly higher levels of these proteins^[129]^. We recently reported that β_1_-AR and β_2_-AR expression and several downstream AR targets were altered in a sex-dependent manner in response to isoproterenol. While expression of β2-AR was several fold increased in aged male hearts in response to AR stimulation, it remained unchanged in the aged female^[119]^. Sex differences have been reported in the activity of adenylyl cyclases and cAMP production^[126,127]^, as well as the activity of phosphodiesterases in cAMP breakdown^[130]^. These studies report that female myocytes have lower levels of basal cAMP. If basal cAMP is lower in females, this would be expected to cause less PKA activation. Indeed, downstream measurements of PKA activity show sex differences, in such that stimulation of β-AR increased Ca^2+^ currents, Ca^2+^ transients and contraction in myocytes from females in comparison to males^[130]^. Testosterone inhibits phosphodiesterase activity in the rat heart^[131]^. The inhibition of phosphodiesterase can potentially explain higher levels of basal cAMP in male hearts compared to females. While these previous findings clearly demonstrate that sex differences exist in the activation of the adrenergic cascade, and that these differences change with age, it is clear that more work is necessary to investigate mechanistic differences in the AR cascade. Given the widespread use of pharmacological agents which target the adrenergic cascade, studies utilizing sex and age as biological variables are needed.

## CONCLUSIONS

Sex differences underlie many facets of cardiac aging, including prevalence, severity and manifestation and susceptibility to a variety of heart diseases. However, despite the observation that men and women age differently (as typified by premenopausal cardioprotection), the mechanisms of sex-specific cardiac aging still remain unclear. Understanding the contributions of sex and age, as well as their complex interplay in the context of cardiac health, represents a fundamental step toward sex-and age-specific medicine and the development of more effective options to prevent and treat heart disease for both male and female patients.

## Figures and Tables

**Table 1.** Common age-related cardiac diseases that exhibit sex-specific differences

	Sex differences	Ref.
Heart failure	HFrEF more prevalent in men HFpEF more prevalent in women Women have more comorbidities, more likely to die with HFpEF	[29,30,31]
Ventricular arrhythmias	More prevalent in men	[32,33]
Ischemic heart disease	Higher risk for development of disease in men at younger age Worse outcomes and higher mortality for women following Ml at older age	[29,35–37]
Valvular disease	Aortic regurgitation more prevalent in men Degenerative mitral valve disease more prevalent in elderly women (> 80 years)	[38]

HFrEF: Heart failure with reduced ejection fraction.

## Data Availability

Not applicable.
